# Genetically Distinct Acute Megakaryoblastic Leukemia following Low Hypodiploid B-Lymphoblastic Leukemia linked by *TP53* Mutation

**DOI:** 10.1177/10935266251316150

**Published:** 2025-02-03

**Authors:** Jaryse C. Harris, Jeffrey Schubert, Brian Lockhart, Rachel Olson, Michele E. Paessler, Elizabeth Margolskee, Vinodh Pillai, Jinhua Wu, Netta Golenberg, Jiani Chen, Elizabeth H. Denenberg, Tammy Luke, Minjie Luo, Yiming Zhong, Marilyn M. Li, Gerald B. Wertheim

**Affiliations:** 1 Department of Pathology and Laboratory Medicine, Hospital of the University of Pennsylvania, Philadelphia, PA, USA; 2 Division of Genomic Diagnostics, Children’s Hospital of Philadelphia, Philadelphia, PA, USA; 3 Department of Pathology and Laboratory Medicine, Children’s Hospital of Philadelphia, Philadelphia, PA, USA

**Keywords:** acute megakaryoblastic leukemia, low hypodiploid B-lymphoblastic leukemia, TP53 mutation, hematopathology, molecular pathology, molecular oncology

## Abstract

We report a case of acute myeloid leukemia with megakaryoblastic differentiation (AMKL) that developed after an initial B-lymphoblastic leukemia (B-ALL) with low hypodiploidy. Although the AMKL was initially thought either to be a phenotypic change from the original B-ALL or to have arisen as a result of treatment (acute myeloid leukemia, post cytotoxic therapy, AML-pCT [WHO]; AML, therapy related [ICC]), genetic evaluation of both the AMKL and the B-ALL suggest that neither of these considerations was correct. Rather, the AMKL did not harbor the most common genetic hallmark of AML-pCT–rearrangement of *KMT2–* and was genetically distinct from the B-ALL. Both the B-ALL and the AMKL, however, showed an identical *TP53* mutation by next generation sequencing (NGS), while germline testing was negative for this mutant allele. Hence, either the patient had a tissue restricted constitutional *TP53* mutation or had a somatic mutation in a multipotent hematopoietic precursor. This case highlights the necessity for close monitoring of patients with *TP53*-mutant tumors, as they may develop multiple lesions despite negative germline testing.

## Introduction

*TP53* is a frequently mutated gene in cancer, particularly in solid tumors^1^; frequency of somatic *TP53* mutations in human malignancies varies between 10% (hematopoietic malignancies) and 96% (high-grade ovarian serous carcinoma).^2,3^ In pediatric leukemia, *TP53* mutations are infrequent and portend poor prognosis.^4-6^

Although most cases of pediatric B-lymphoblastic leukemia (B-ALL) lack mutations in *TP53*, over 90% of B-ALL with low hypodiploidy (defined by a total of 32-39 chromosomes) have *TP53* alterations, and about half of these have germline *TP53* mutations (i.e., Li-Fraumeni syndrome).^7-9^ This close association necessitates evaluation of *TP53* status in normal tissue from patients with B-ALL with low hypodiploidy to assess their risk of additional tumors and to establish potential risk for family members. Additionally, low hypodiploid B-ALL is associated with high-risk disease regardless of age,^10^ likely due to *TP53* mutation.^11,12^

## Description of Case

Here we discuss a case of a young adult female patient with a history of very high-risk low hypodiploid B-ALL with mutated *TP53* who subsequently developed acute myeloid leukemia, best characterized immunophenotypically as acute megakaryoblastic leukemia (AML with mutated *TP53* by 2022 International Consensus Conference criteria).^13^

**Figure 1.**
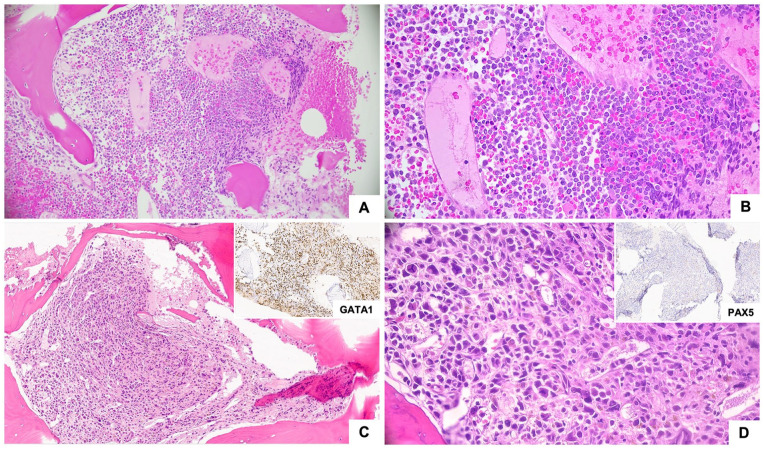
Low (50×, A and C) and high (400×, B and D) magnification photomicrographs of bone marrow biopsies. B-ALL (Panels A and B) demonstrates sheets of uniform immature cells with high N:C ratio. AMKL (Panels C and D) shows sheets of large pleomorphic cells with variable nuclear size that are GATA1-positive (inset C), and PAX5-negative (inset D).

**Figure 2.**
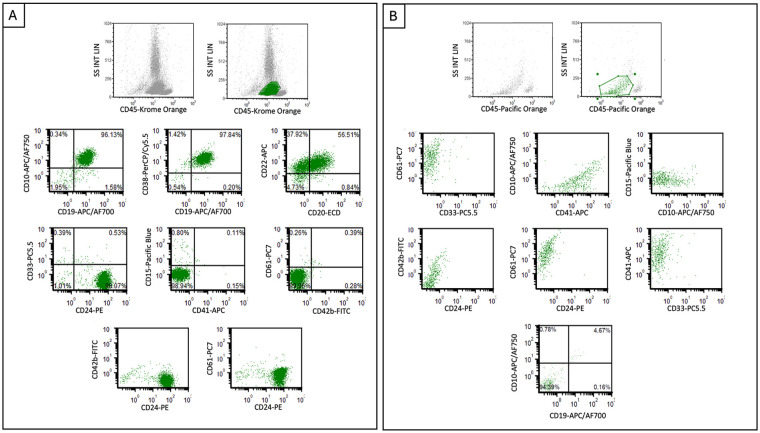
Flow cytometric plots from diagnostic samples. B-ALL (Panel A) shows CD45(dim) blasts positive for CD20, CD22, CD19, CD24, CD10, and CD38, while negative for CD42b, CD61, and CD41. AMKL (Panel B) shows blasts positive for CD41 and CD61 with dim partial CD42b expression, while negative for lymphoid markers including CD19, CD10, and CD24.

**Figure 3.**
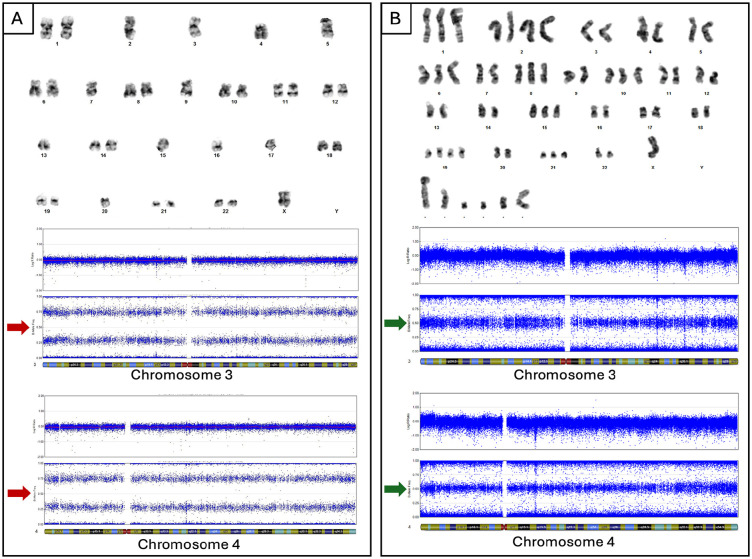
Representative images of karyotypes and SNP arrays of diagnostic samples. B-ALL (Panel A) karyotype shows monosomy of chromosomes 2, 3, 4, 5, 7, 9, 13, 15, 16, 17, 20, and X. SNP analysis (chromosomes 3 and 4) confirms chromosomal loss with loss of heterozygosity by B allele fraction (red arrows). AMKL (Panel B) karyotype shows near-triploid clone. SNP analysis (chromosomes 3 and 4) indicates retention of heterozygosity (green arrows), confirming tumors are not clonally related.

**Figure 4.**
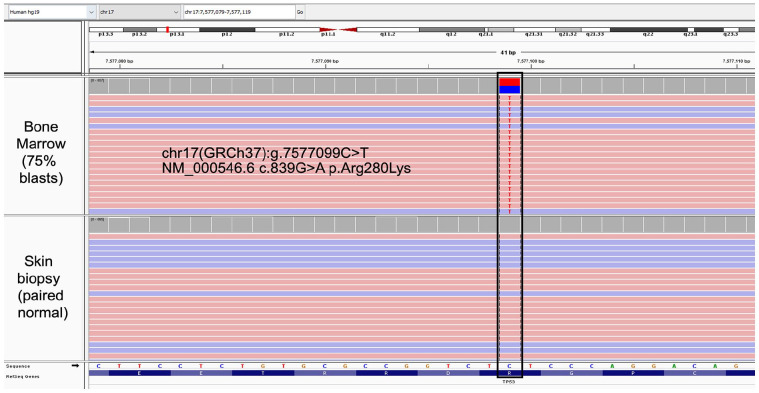
Next Generation Sequencing with integrative genome viewer from B-ALL sample reveals TP53 c.839G > A (p.Arg280Lys) mutation in bone marrow specimen with 75% blasts, absent in skin sample.

The patient, a 20-year-old female, presented with jaw and neck discomfort and was treated with NSAIDs and antibiotics for temporomandibular joint dysfunction and lymphadenitis. Persistent symptoms and onset of night sweats prompted further evaluation. Physical exam revealed hepatosplenomegaly and cervical lymphadenopathy. Head and neck CT scans showed enlarged cervical and submandibular lymph nodes (largest 1.3 cm). Labs showed elevated lactate dehydrogenase (1176 U/L, reference range: 313–618 U/L), and CBC showed anemia (hemoglobin 6.7 g/dL, reference range: 12.0–16.0 g/dL) thrombocytopenia (17 k/µL, reference range: 150–400 k/µL) and blasts on peripheral smear (20% outside hospital, 18.2% on day of marrow). Bone marrow morphology (Figure 1) and flow cytometry (Figure 2(a)) confirmed B-ALL with 75% of blasts on aspirate that were positive for CD45(dim), CD10, CD19, CD22, CD9, CD20(subset), CD24, CD34, CD38, cCD79a, HLA-DR, and TdT (64% of total events), while negative for T-cell and myeloid markers (including megakaryocytic markers, CD42b, CD61, and CD41). Genetic studies (karyotype; FISH; and NGS for mutations, copy number variations, and common fusions) demonstrated a low hypodiploid genome (total of 35 chromosomes) with losses of chromosomes 2, 3, 4, 5, 7, 9, 13, 15, 16, 17, 20, and X (Figure 3), and a mutation in *TP53* c.839G > A (p.Arg280Lys) at an allele fraction of 54% (Figure 4). A diagnosis of low hypodiploid B-ALL was rendered. Germline testing was performed on a skin biopsy and was *TP53* wild type (Figure 4).

The patient started COG AALL1732 treatment protocol with intrathecal cytarabine and induction therapy (vincristine, daunorubicin, and prednisone). Bone marrow showed 1.43% of mononuclear cells were residual blasts by flow cytometry at the end of induction (very high risk by measurable residual disease at the end of induction^14^), and by 2 months, marrow evaluation including flow cytometry showed no residual disease. Treatment continued with asparaginase, methotrexate, cyclophosphamide, mercaptopurine, and inotuzumab; however, at day 78 after initial diagnosis/induction, she re-presented with cytopenias and menorrhagia requiring transfusions. Peripheral blood findings showed neutropenia (1300/µL, normal 2000–7150/µL), anemia (11.3 g/dL), and thrombocytopenia (10 k/µL). An aparticulate bone marrow study demonstrated 15% blasts by cytology. The concomitant biopsy had sheets of blasts with a few hypolobated megakaryocytes (Figure 1). Flow cytometry did not show an expansion of lymphoblasts (note lack of CD19, CD10, and CD24 staining, Figure 2(b)), yet did show 20% of events were positive for CD41 and CD61. Further immunohistochemical evaluation of the biopsy revealed numerous blasts positive for CD42b and GATA1, confirming megakaryocytic differentiation (Figure 1(c) inset image showing GATA1 positivity). The majority of blasts were negative for PAX5, CD79a, CD3, MPO, TdT, and CD34.

Given the relatively low number of blasts on the aspirate—perhaps due to peripheral blood contamination—repeat bone marrow study was performed, showing 78% blasts and adequate material for genetic evaluation. Results showed a hyperdiploid (near triploid) genome with gain of 1 or 2 copies of chromosomes X, 1, 2, 6, 10, 14, 15, 19, 20, 21, 22, and multiple different marker chromosomes (Figure 3). The identical *TP53* mutation identified in the initial B-ALL yet absent in the normal skin specimen, was also observed (variant allele frequency 90%). Cytogenetics and Archer fusion sequencing were negative for *KMT2A* rearrangement or other rearrangements associated with AML-pCT. These findings initially suggested clonal evolution and phenotypic switch of the B-ALL; however, as chromosomes that were lost in the B-ALL were clearly present in the AMKL (Figure 3), this is not an appropriate explanation. Thus, the tumor does not have the genetic or phenotypic hallmarks of AML-pCT and is clonally unrelated to the B-ALL.

The patient started AML therapy, but rapidly deteriorated with sepsis, acute pancreatitis, acute respiratory distress syndrome, and acute renal failure requiring continuous renal replacement therapy. She ultimately developed refractory shock secondary to *Pichia kudriavzevii* fungemia. She passed away on day 110 post-AMKL diagnosis.

## Discussion

We described a patient with 2 genetically and immunophenotypically distinct leukemias that demonstrated the same *TP53* mutation. This case highlights the importance of careful monitoring in patients with leukemia harboring a *TP53* mutation, irrespective of germline status. In comparison to many solid tumors, *TP53* mutations are less frequent in ALL, except in cases of relapsed disease and low hypodiploid B-ALL, both of which carry poor prognoses.^15^ *TP53* mutations are also rare in pediatric AML.^16^ AMKL is a rare leukemia in adults (<1% of cases) and comprises between 4% and 15% of AML in pediatric patients.^17^ Adult AMKL frequently carries *TP53* mutations, while pediatric cases are more likely to have recurrent genetic fusions.^18,19^ Recognizing *TP53* mutations is important in hematologic malignancies for prognostic and therapeutic purposes.^20,21^

*TP53* mutations have been documented to be early events in development of low hypodiploid B-ALL either as inherited (Li-Fraumeni)^15^ or as pre-leukemic somatic mutations.^22^ In either case, cells possessing these mutations may persist following successful therapy of initial B-ALL. Therefore, even if patients with low hypodiploid B-ALL do not carry a germline *TP53* mutation, they should be monitored for both relapse of initial B-ALL and for a genetically discrete solid or hematopoietic neoplasm.

This case raised several possibilities for the oncogenesis of the second hematologic malignancy (AMKL after treatment for B-ALL). One possibility considered, though highly unlikely, was that the identical *TP53* mutations identified in the AMKL and B-ALL arose from 2 independent mutational events. The second possibility was that the patient had germline tissue-specific, mosaic *TP53* mutation absent in skin tissue assessed for germline alterations. The third possibility was that an early multipotent hematopoietic cell acquired a *TP53* mutation. These latter 2 possibilities are somewhat related and may be difficult to distinguish.

Initially, a phenotypic switch from B-ALL to AMKL was considered since at the time of relapse a small lymphoblast population (3.1%) and AMKL were seen in the marrow. In light of genomic evaluation, wherein the B-ALL had loss of multiple chromosomes (e.g., 2, 3, 4, and X) that were clearly present in the AMKL, this possibility was excluded (Figure 3). The lymphoblasts were favored to be hematogones. Additionally, the B-ALL was positive for an IgH gene rearrangement by B cell clonality, and the AMKL did not show the IgH gene rearrangement despite an adequate DNA sample. Given the cytotoxic therapy and timing of relapse, a *KMT2A* rearrangement was also considered; however, this alteration was not identified nor were other alterations associated with AML-pCT. Likewise, the timing of relapse and lack of dysplasia, makes therapy-related disease without *KMT2A* rearrangement unlikely.

Altogether, the B-ALL and AMKL in this patient appear to be 2 completely different primary tumors that are genetically linked by the identical *TP53* mutation. Either tissue restricted germline mosaicism or somatic mutation in an early undifferentiated hematopoietic precursor is the most probable explanation. Distinguishing these 2 possibilities would be academic as the patient would be at risk for multiple tumors regardless of *TP53* mutation origin. Given this case of 2 *TP53*-mutated malignancies, we would recommend close follow up for young patients with mutations even in the absence of documented germline mutations.
